# Usability testing of a user-centered clinical decision support tool about asymptomatic bacteriuria and urinary tract infections in older adults

**DOI:** 10.1017/ash.2026.10826

**Published:** 2026-08-28

**Authors:** Yosef Chernet, Mary C. Politi, Viktoria Vonder Haar, Kevin Hsueh, Eliana Charlotte Goldstein, Sena Sayood, Michael J. Durkin

**Affiliations:** 1 Washington University School of Medicine, St Louis, USA; 2 School of Public Health, Washington University, St Louis, USA; 3 Division of Infectious Diseases, Washington University School of Medicine, St Louis, USA

## Abstract

**Objective:**

We evaluated usability of a web-based clinical decision support tool designed to support antibiotic stewardship by helping clinicians differentiate asymptomatic bacteriuria from true urinary tract infections.

**Design:**

Mixed methods usability evaluation with qualitative think-aloud sessions and survey.

**Setting:**

Private academic medical center.

**Participants:**

Clinicians who evaluate and treat older adults with suspected asymptomatic bacteriuria or urinary tract infections.

**Methods:**

Participants used the tool to assess three standardized clinical scenarios, verbalizing their thoughts as they used the tool, followed by a short debrief interview and survey. Transcripts of audio recordings were analyzed using qualitative content analysis across four usability domains: navigation, content, functionality, and layout.

**Results:**

Seven clinicians (four attending physicians, three advanced practice providers) practicing in primary care, outpatient, and inpatient settings participated. The mean System Usability Score was 85.4 (SD = 10.5; range 75–100), indicating “excellent” usability. Three qualitative themes emerged: (1) positive responses to the tool’s speed, simple entry, and reassuring guidance; (2) content-related challenges, including missing symptoms, unclear fever thresholds, undefined terminology, and absent caveats for allergies, resistant organisms, or unique populations; and (3) suggested navigational changes, including links opening in new tabs, preserving entered clinical data, and reorganizing the symptom checklist. Participants highlighted electronic health record integration as necessary for sustained use.

**Conclusions:**

Participants found the tool usable and clinically valuable, particularly when infectious disease expertise is limited. Targeted refinements will be addressed before testing in a randomized study with a larger more representative sample. Future work will also pursue electronic health record integration via an embedded hyperlink for broader deployment.

## Introduction

Asymptomatic bacteriuria (ASB) is a benign condition where one has the presence of bacteria in urine without symptoms of a clinical infection. Older adults are commonly prescribed antibiotics for ASB; clinicians often fail to distinguish ASB from a true urinary tract infection (UTI) in older adults given non-specific symptoms of aging (eg, more frequent urination in general or mild cognitive changes).^1–3^ Patients with ASB may account for almost half of UTIs diagnoses in older female adults.^4^ Older adults and their caregivers may also request antibiotic therapies due to prescribing behaviors of previous clinicians,^1^ online misinformation about UTIs, with numerous websites and AI-generated summaries reporting urinary changes (eg, color, odor) that do not necessarily indicate a UTI, or concern about symptoms worsening.^5^ Overprescribing antibiotics can accelerate antimicrobial resistance^6,7^ and adverse drug effects.

Clinical decision support (CDS) tools can improve quality, consistency, and safety of care by providing evidence-based recommendations to clinicians.^8–10^ CDS tools can help clinicians distinguish between UTIs and ASB in older adults, yet few are incorporated into routine practice. We previously evaluated the feasibility, appropriateness, and sustainability potential of CDS tools that help clinicians distinguish between UTIs and ASB in older adults.^1,11,12^ We also developed patient-facing materials aimed at improving patient and caregiver knowledge and engagement to incorporate into a CDS tool.^1^

In order for CDS tools to be used in routine practice and supported by clinicians, they should be fast, functional, and easily integrated within existing workflows.^13^ This paper describes usability testing of a UTI CDS tool to gather data on strengths in function and use and identify areas for improvement prior to routine implementation.

## Methods

### Participants and recruitment

Eligible participants were clinicians who work at an academic medical center in the Midwest or a community partner site, who evaluate and treat patients with suspected asymptomatic bacteriuria or urinary tract infections. Participants were recruited via publicly available email addresses and via snowball sampling of enrolled participants.^14^ Recruitment was ended after interviews were no longer producing new usability issues. All participants reviewed a consent information sheet prior to the interview. The study was approved as exempt research by the Washington University Institutional Review Board (#202406156).

### Procedures

#### Think-aloud usability sessions

**Figure 1.**
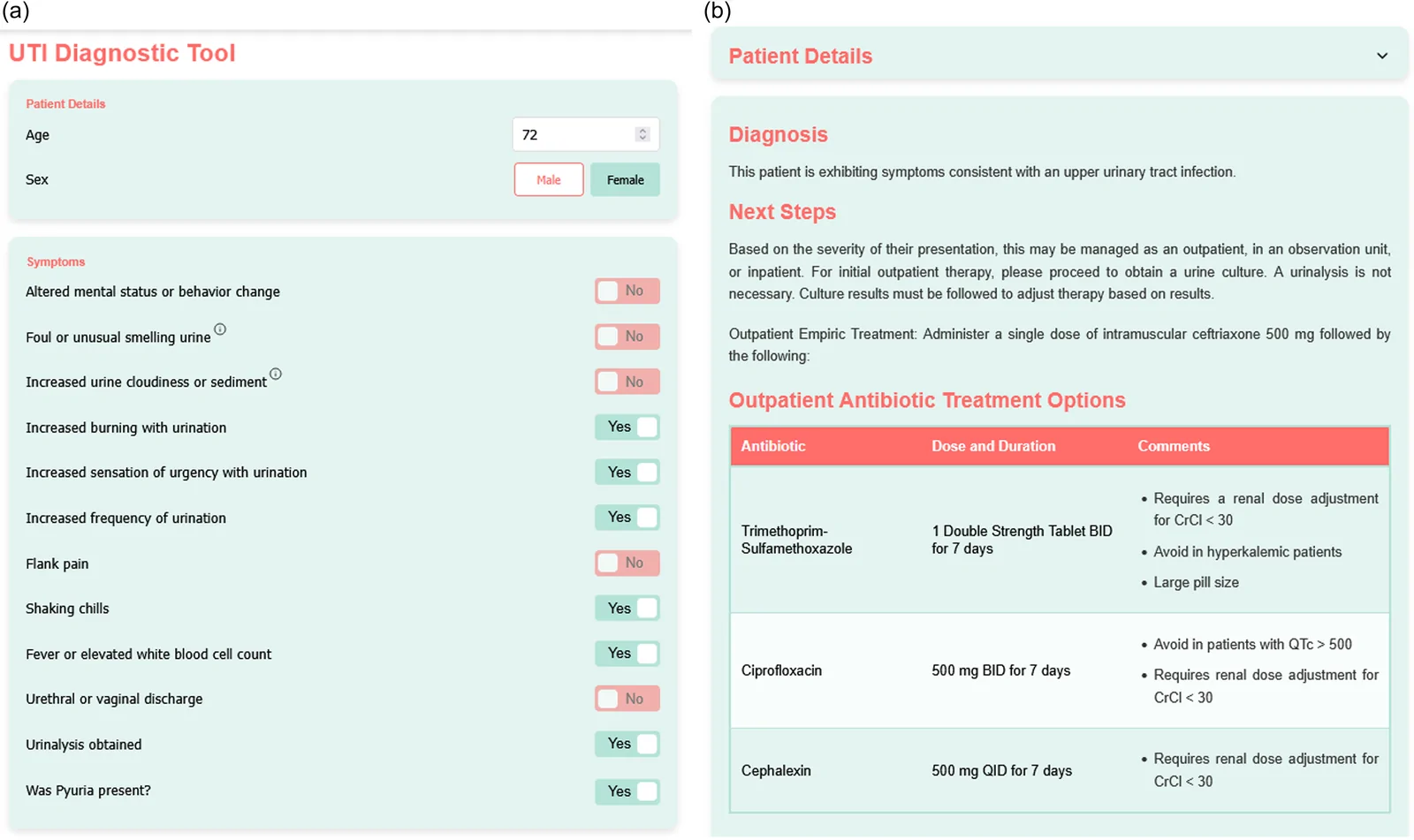
Screenshots of the web-based clinical decision support tool. (a) The input page, with sample patient details and symptoms from a clinical scenario included in usability testing. (b) The output page based on the provided details and symptoms. Figure 1 long description.

We conducted a focused usability evaluation of a beta version of a UTI CDS tool for older adults. The tool is a web-based application with interactive elements (see Figure 1a and b). It includes yes or no toggle options for symptoms, text entry and arrow-based inputs for age, and a selectable button for sex assigned at birth. Once the clinical data are entered, the tool provides appropriate recommendations for possible UTI, ASB, or other differential diagnoses. The recommendation page also cites relevant guidelines for management of the corresponding diagnosis.

First, a team member (VVH) conducted a structured, moderated think-aloud usability session.^15–17^ We gave participants three clinical scenarios to enter into the UTI CDS to simulate common real-world clinical cases. Scenarios were reviewed prior to the start of data collection by a clinical advisory panel of three practicing clinicians with expertise in antimicrobial stewardship, including for urinary symptoms. Each session began with a brief orientation and then a series of predefined tasks, such as reviewing symptoms and risk factors, assessing eligibility for treatment, selecting an antibiotic and duration, and documenting a plan for next steps.

Participants were instructed to verbalize their thoughts while completing the tasks. The moderator used a standardized guide (see Supplementary Materials), prompted participants to explain their thoughts when needed (ie, if the participant was silent for >10 s while completing a task), and avoided coaching except when a critical usability issue prevented task completion. Sessions concluded with a short debrief session to capture overall impressions of the CDS tool, perceived usefulness, workflow fit, and suggested changes. Sessions were audio and video recorded and transcribed for analysis.

#### System Usability Scale (SUS) survey

After completing usability testing of the CDS tool, participants completed the System Usability Scale (SUS),^18^ a 10-item survey that produces a composite usability score from 0 to 100, with higher scores indicating better perceived usability.

#### Demographic survey

Participants reported their clinical role, years in practice, and primary practice setting in a brief survey at the end of the session.

### Data analysis

#### Qualitative analysis

Think-aloud transcripts and debriefing responses were analyzed using qualitative content analysis.^19^ Analyses uncovered usability problems, workflow barriers, and design recommendations. We assessed four a priori usability domains based on a framework used in past work:^15,16^ (1) navigation; (2) terminology and clinical content clarity; (3) functionality, visibility, and completeness of diagnostic recommendations; and (4) interface layout. An initial codebook was developed deductively from established usability frameworks and then inductively from early transcripts. Coded segments were organized into thematic clusters, including symptom checklist completeness and labeling; antibiotic recommendation accuracy and safety; algorithm logic errors; reference link accessibility; navigation and data persistence; and perceived clinical utility across clinician types and care settings. Findings were synthesized into a prioritized list of actionable design recommendations, with themes supported by representative participant quotes. Two members of the research team (YC, VVH) reviewed a subset of transcripts to establish coding consistency, and discrepancies were resolved through discussion.

#### Quantitative analysis

SUS scores were calculated per standard scoring procedures,^18^ in which alternating items are reverse-scored, and totals are multiplied by 2.5 to yield a 0–100 scale. A score ≥68 is considered above-average usability. We calculated individual and mean SUS scores and summarized results with descriptive statistics (mean, standard deviation, range). Item-level responses were examined to identify specific usability dimensions such as ease of use, consistency, and need for technical support that warranted targeted improvement.

## Results

### Participants

Ten eligible clinicians were invited to participate through publicly available email based on inclusion criteria and clinician referral. Of these, seven (70%) responded and agreed to participate. All seven participants who enrolled (100%) completed their usability sessions.

Participants included four attending physicians (57%) and three advanced practice providers (43%). All participants (100%) reported practicing in a primary care or outpatient setting, and three (43%) reported that they also practice in an infectious disease inpatient setting. Participant characteristics are summarized in Table 1.

**Table 1.** Usability interview participant characteristics (N=7) Table 1 long description.

Characteristic	N=7
**Clinical role, n (%)**	
Attending physician	4 (57%)
Advanced practice provider	3 (43%)
**Years in practice, n (%)**	
0–2 years	1 (14%)
3–5 years	2 (29%)
6–10 years	2 (29%)
10 years or more	2 (29%)
**Primary practice or setting, n (%)** ^a^	
Inpatient ward	3 (43%)
Primary care/outpatient	7 (100%)

a Participants could select more than one primary practice setting; percentages do not sum to 100%.

### Qualitative outcomes: think-aloud sessions

We identified three key themes from the think-aloud section and documented related suggestions. Table 2 summarizes key suggestions with sample quotes from participants.

**Table 2.** Suggestions from think-aloud sessions with sample supporting quotes Table 2 long description.

Theme	Example quotation
**Participants provided content suggestions to improve the UTI CDS tool’s usability.**	
Suggested adding some missing symptom options	“…it didn’t mention at least that I saw any mention of costovertebral, tenderness or anything like that. A lot of people I know consider that as a telltale sign for an upper [Urinary] tract infection.” (Participant 005)
Recommended adding a definition of a fever threshold	“I’m gonna say that this is not a classic fever [100.2°F, which was not considered fever by the tool], but this is fever for a 72-year-old. I would actually take that seriously.” (Participant 003)
Some terminology not universally used; provided input on clarity of language and possible overlapping terms	“I don’t actually think about upper and lower urinary tract infections when I think about UTIs, but I probably should…I think of complicated and uncomplicated UTIs.” (Participant 006)
Suggested adding an allergy caveat adjacent to antibiotic recommendations	“You don’t want to act like it’s recommending just to give Bactrim and not have something in there referencing allergies” (Participant 002)
Recommended information about special patient populations	“If your patient is on chemotherapy or is a renal transplant patient…the recommendations for shorter antibiotic durations… you can’t necessarily extrapolate to that” (Participant 002)
Requested more background information and resources to review guidelines	“Having a link somewhere to a website that gives dose adjustments for creatinine clearance would be helpful… Like, underneath here [Outpatient Antibiotic Treatment Options], it could just be a small link, if those just as needed, you can click here.” (Participant 001)
**Participants provided suggestions to the UTI CDS tool’s navigation and layout to improve usability.**	
Entered data was lost when navigating back from an external resource; suggested fixing the navigation of resources by having links open in a new tab	“…the last one, when we did it, it [the recommendations page] opened up in the same window and closed out your algorithms, so it got rid of the suggestions.” (Participant 001)
Symptom checklist not organized alphabetically or by category; suggested making it easier to find symptoms	“It’d be nice if these were ordered, maybe alphabetically, so that you don’t have to hunt for them.” (Participant 002)

UTI, urinary tract infection; CDS, clinical decision support.

### Theme 1: Overall, participants had positive responses to the UTI CDS tool

All participants expressed enthusiasm for the UTI CDS tool’s potential to support prescribing decisions in older adults, particularly in settings where UTI over-treatment is common. One participant noted, *“I can see this being extremely helpful in nursing homes… primary care… people that aren’t as familiar with diagnosing and treating UTIs”* (Participant 003).

Clinicians appreciated the tool’s speed and interface design. Providers noted that CDS tools are abandoned when they feel slow or cumbersome, and the UTI CDS tool avoided this pitfall by generating recommendations quickly and presenting them in a clean, uncluttered layout. As one participant said, *“It’s quick. The worst thing that you do is you fill out all these things and then you click a button it takes 10 s to load. No one wants to wait that long.”* (Participant 002) The yes/no toggle format for symptom entry was considered an effective design choice, valued for keeping the interaction objective and focused. As one participant put it, there is *“not a lot of room to kind of go off on a tangent. It’s just yes or no, and…stays very objective”* (Participant 003).

The clinical content of the tool’s recommendations was considered “reliable” (Participant 006). Participants with infectious disease backgrounds noted that the outputs aligned with their own clinical reasoning. The ASB guidance was described as reassuring and would help clinicians feel comfortable with a decision to actively monitor rather than treat non-specific urinary symptoms, even under patient or caregiver pressure. As one participant stated, *“okay, you’re not just… being negligent [By avoiding antibiotics for ASB], this is the guideline, like, you’re okay” (Participant 007).*

### Theme 2: There were some identified usability challenges with the UTI CDS Tool

#### Content

Content-related findings were the most substantive category of the usability evaluation, encompassing the symptom checklist, the tool’s algorithm logic, and its antibiotic recommendations. These issues generated the most unprompted engagement across sessions and, where present, were associated with the highest levels of participant feedback.

#### Symptom list and clarifications

A consistent theme across sessions was that several elements of the symptom checklist required clarification or expansion. This is particularly relevant for those working outside of infectious disease or in settings where UTI’s and ASB are frequently encountered. Issues ranged from missing symptom options to undefined clinical terms.

For example, the absence of lower abdominal pain and costovertebral angle tenderness as selectable symptoms was an important point mentioned. Participants scanning the checklist for this common symptom of cystitis found only flank pain listed, and the distinction between the two was clinically meaningful. Clinicians felt that they needed to select an imprecise alternative or simply omit the symptom, potentially affecting the tool’s recommendation.

Clinicians felt the fever item required clarification given the intended use with older adults. Older adults often mount a blunted febrile response to serious infection, meaning a temperature that does not meet standard fever criteria may nonetheless be significant in an older adult.^20^ As one participant noted, a temperature of 100.2 °F *“could be high for an old person who doesn’t have a fulminant immune response”* (Participant 006), while another pointed out that in nursing home settings, staff may report any elevation above baseline as a “fever” regardless of threshold.

Several terms used in the tool’s output were not universally understood. The phrase “localizing symptoms” appeared without inline definition, leaving those unfamiliar with the term uncertain about which of their patient’s symptoms qualify. Similarly, the classification of infections as “upper” versus “lower” urinary tract was not familiar to all participants, with some more accustomed to the terminology of complicated versus uncomplicated UTI.

#### Antibiotic recommendations

The antibiotic recommendation section raised some concerns; these issues were notable because it is one of the most important actionable elements of the tool, and any errors or omissions carry clinical consequences.

The absence of any antibiotic allergy caveat was identified as a patient safety gap. The tool presented empiric antibiotic options without any prompt to cross-reference the patient’s documented allergies or medication history. As one participant observed, some users may approach the tool’s output as a near-direct prescription rather than as a starting point for further clinical judgment: *“Others might use this as an immediate decision tool without additional critical thinking”* (Participant 002).

Several additional individual-level factors were identified as missing considerations that could affect antibiotic selection. A history of resistant UTI organisms was raised as clinically important information that the tool does not currently capture. The absence of any caveat for immunocompromised patients was also noted: current complicated UTI guidelines explicitly exclude this population from standard duration recommendations, meaning the tool’s outputs could be misapplied.

Finally, given the high prevalence of impaired renal function in older adults, a link to renal dose adjustment resources adjacent to the antibiotic section was described as a practical and high-value addition that would support safe prescribing without expanding the tool’s core scope.

### Theme 3: Participants discussed navigational and layout changes that could improve the UTI CDS tool’s usability

#### External reference links opening in the same browser tab

The most consistently raised navigation concern was that certain external reference links opened within the same browser tab, rather than in a new tab, causing the current session to be lost or interrupted. As one participant stated: *“I just clicked that link, and then I tried to go back and it reset it. So I cannot go back to my conclusion. So maybe make sure it should open in a different tab.” (Participant 4).* Other participants similarly found themselves disoriented after following a link, unable to locate the tool again without interviewer assistance.

#### Loss of entered data when navigating back

A related concern was that using the browser back button to return to the symptom input page cleared all previously selected entries, requiring re-entry of patient data. Several participants were frustrated by this process and recommended adjusting the tool such that data could be saved rather than reset. While one participant acknowledged that a clean slate on returning might be intentional in some workflows, most favored storing data entry to support iterative review, similar to other clinical decision support.

#### Organization of the symptom checklist

Several participants suggested that the symptom checklist would benefit from being organized either alphabetically to reduce the time needed to locate specific items or in separate categories. One (P002) noted having scanned the list twice before concluding that abdominal pain was not present as an option, for example.

### Quantitative outcomes

The mean SUS score across all seven participants was 85.4 (SD = 10.5), exceeding the established threshold of 68 for above-average usability and falling within the “Excellent” rating on the SUS.^21^ All seven participants scored above the acceptability threshold with scores ranging from 75 to 100. *Debrief Outcomes*

During debrief sessions, Electronic Medical Record (EMR) integration was raised by several participants as a possible prerequisite for meaningful adoption. Even as the CDS tool was recognized as clinically valuable, many participants had concerns about whether it would be used, *“One of the biggest usability problems with any of these decision assistance tools is you have to have it… it has to be in a place where you don’t have to look for it”* (Participant 002).

## Discussion

This study explored the usability of a UTI CDS tool for use in routine care. Overall, the UTI CDS tool was received positively. Participants supported the main goal of the tool which is to mitigate antibiotic overprescribing in older adults with non-specific urinary symptoms. Participants recognized the scope of the problem and possible role of the tool in filling a clinical need. They described the tool as well-suited to contexts where clinicians may lack the experience or confidence to navigate the distinction between UTI or ASB, or to recognize presentations that warrant a broader differential.

The tool’s design elements like fast load time, binary data entry, and a clean recommendation output were appreciated as facilitators for use. CDS tools face adoption challenges if they are slow, cognitively demanding, or disruptive to workflow, regardless of their clinical validity.^22^ The speed and ease of use of the tool was mentioned positively across multiple sessions, suggesting it occupies an important position in participants’ mental model of what makes a CDS tool viable in practice.

Another potential benefit was the tool’s ability to reassure clinicians about their decisions. Antibiotic stewardship guidance is sometimes framed as a way to reduce over-treatment by improving clinician knowledge. However, even when clinicians are familiar with the evidence, they might hesitate to withhold antibiotics in some situations where there is uncertainty or patient request. By explicitly citing guidelines, a CDS tool can address a need beyond informational accuracy.

While participants were broadly supportive of the tool, the think-aloud sessions revealed numerous suggestions that can inform revisions to enhance usability and patient safety. For example, the symptom checklist assumed a degree of clinical familiarity that not all intended users might have. Clarifying and organizing symptom lists could address this issue. The antibiotic recommendation section also raised some concerns about the absence of a caveat for potentially allergic or immunocompromised patients. The value of CDS tools is partly realized through the safety nets they create, such as alerts that catch errors that individual clinical judgment may miss under time pressure.^23^ Adding information about antibiotic safety could improve the tool’s functionality.

Navigation and layout feedback pointed to issues like the loss of entered data when navigating back from external links, which can be frustrating. In a clinical environment where time is scarce and interruptions are frequent, a tool that requires full re-entry of information after a single navigation misstep may erode user confidence.

Concerns about EMR integration were widely expressed across sessions and reflect a well-documented challenge in the CDS literature, namely that adoption of CDS tools is determined as much by access as by quality.^24^ Participants also suggested a concise, plain-language summary of the main recommendations, included in-line with the full citations, to better support decision-making during patient care.

### Limitations

Several limitations of this study should be acknowledged. The small sample from a single region, while appropriate for a think-aloud usability study of this type,^25^ may not represent all clinician views. The perspectives represented, though clinically diverse, may not fully capture the range of clinician backgrounds and practice contexts in which the tool would be used. Nursing home staff and pharmacists working in community settings, for example, were not represented in this sample. In addition, standardized clinical scenarios are essential for consistency but differ from real clinical settings. Participants used the tool in a controlled research setting, knowing they were observed and that their feedback was the main goal. This could lead to slower and more focused use of the tool than in busy clinical situations. The lack of patients, who influence the pace, focus, and emotional tone of clinical interactions, is a natural limitation of laboratory-based usability testing. Finally, the think-aloud protocol captures the usability experience of first-time or early users. It does not address how perceptions of the tool might shift with repeated use, how the identified issues would affect clinical outcomes over time, or how the tool would perform across the full patient population it is designed to serve. Longitudinal evaluation of the tool’s impact on prescribing behavior and, ultimately, on patient outcomes will require a separate phase of study following the refinements identified here.

Overall, this study highlights the importance of conducting usability evaluations during the development of CDS tools before they are widely deployed. Think-aloud usability testing is particularly well-suited to this stage of development. The sessions produced feedback that would not have been captured by a postuse survey or a quantitative usability metric. Based on the findings of this usability testing, our prototype UTI CDS tool may be acceptable and important for use. However, several revisions to the CDS tool will be addressed before additional testing in larger studies and broader deployment in routine care.

## Supporting information

10.1017/ash.2026.10826.sm001 Chernet et al. supplementary material Chernet et al. supplementary material

## Data Availability

Deidentified transcripts available from the authors upon reasonable request.

## Supplementary material

The supplementary material for this article can be found at https://doi.org/10.1017/ash.2026.10826.

## Figure 1. Long description

A screenshot of an interactive web tool that allows the input of patient details and symptoms. Patient details are age, entered as a number, and sex, for which clinicians can select male or female. The symptoms include altered mental status or behavior change; foul or unusual smelling urine; increased urine cloudiness or sediment; increased burning with urination; increased sensation of urgency with urination; increased frequency of urination; flank pain; shaking chills; fever or elevated white blood cell count; urethral or vaginal discharge; and whether or not urinalysis was obtained and if pyuria was present. The symptoms are input with yes/no toggles. The tool is displaying sample inputs for a 72-year-old female with burning, urgency, and increased frequency of urination; shaking chills; fever; and pyuria. A screenshot of the diagnosis, next steps, and outpatient antibiotic treatment options that would be output based on the details in the first screenshot. The diagnosis is an upper urinary tract infection. The next step includes urine culture followed by intramuscular antibiotics and an antibiotic from the treatment option table. The treatment option table includes columns for antibiotic name, dose and duration, and comments related to prescribing considerations.

Navigate back to Figure 1..

## Table 1. Long description

The table describes participant characteristics, including their clinical role (attending physician or advanced practice provider), years in practice, and typical practice setting (inpatient or outpatient).

Navigate back to Table 1..

## Table 2. Long description

The table includes quotes related to three themes that emerged from qualitative analysis of usability testing. The first theme was: “Overall, participants had positive responses to the UTI CDS tool.” The second was: “Participants provided content suggestions to improve the UTI CDS tool’s usability.” The last theme was: “Participants provided suggestions to the UTI CDS tool’s navigation and layout to improve usability.”

Navigate back to Table 2..
