# Supplementary material for: Usability testing of a user-centered clinical decision support tool about asymptomatic bacteriuria and urinary tract infections in older adults
Source: Antimicrob Steward Healthc Epidemiol. 2026 Aug 28;6(1):e241. doi: 10.1017/ash.2026.10826 (PMC13760293; doi:10.1017/ash.2026.10826)
Supplement: Supplementary file 1 [file S2732494X26108262sup001.docx]

**UTI CDS Tool Interview Guide**

Thank you for taking the time to speak with us. Our goal is to understand how clinicians like you use the UTI Decision Tool and identify opportunities to improve it, so we can make it as useful as possible.

During this session, I’ll ask you to share your screen and begin using the tool. Based on the two patient information sheets I’ve provided, please imagine you are using the tool to support your clinical decision-making for diagnosing and managing the patients. As you use the tool, I’d like you to think aloud by sharing what’s on your mind from the moment you open the tool. Don’t worry about phrasing things perfectly. If you’re sil+ent for too long, I may ask a question to help prompt your thinking. You’ll have about five minutes to review a patient scenario, use the tool, and generate a recommendation before moving to the next one. There’s no need to rush. Once you’ve completed all the scenarios, I will ask you some questions to reflect on your experience, and then I’ll ask you some survey questions. Do you have any questions before we begin?

[Pause]

*Probes: To be asked as appropriate based on the participant’s think-aloud response. We will only use if they stop thinking out loud for more than 20-30 seconds.*

- General
- If they stop clicking/writing/etc. for more than a few seconds
- What are you thinking about right now?
- Is there something you’re stuck on? [if yes: What is it?]
- If they seem to be struggling with a task—expressing nonspecific frustration, trying to do the same thing repeatedly, etc.
- Is there anything you’re finding difficult or frustrating right now?
- What is making this task difficult?
- Is there information that would be helpful about how to do this task?
- Age entry
- Content
- If the participant pauses or becomes frustrated while selecting symptoms:
- Do you find the list of symptoms to be adequate?
- Do you find that all the symptoms are clear and distinct?
- What changes would you make to the symptom list?
- Is it easy to distinguish between selecting “yes” or “no”

[when the participant completes all case scenarios] Okay, we’re done!

**Debrief**

Now, I have a few questions about your experience with the decision tool.

- How do you feel about the tool overall?
- What aspects of the tool worked well?
- Were you able to find the information you needed quickly?
- How reliable do you find the information in the tool?
- What aspects of the tool were challenging or frustrating?
- What aspects of the tool would you change to improve the tool’s usability?
- What aspects of the tool would you change to fit with your workflow?
- Do you have any additional comments about how this tool might affect antibiotic prescribing for asymptomatic bacteriuria or suspected urinary tract infections in older adults?

Thank you for your thoughts! I’m going to stop recording now to ask you some survey questions.
